# Impaction of Deciduous and Permanent Teeth Related to Local Obstacles: A Retrospective Study of 10 Years of Institutional Experience

**DOI:** 10.3390/children12070929

**Published:** 2025-07-14

**Authors:** Luisa Limongelli, Giuseppe Barile, Giusy Fanelli, Tommaso Corsalini, Saverio Capodiferro, Massimo Corsalini

**Affiliations:** Department of Interdisciplinary Medicine, University of Bari “Aldo Moro”, Piazza Giulio Cesare, 11, 70124 Bari, Italy; luisa.limongelli@uniba.it (L.L.); giusinafanelli@gmail.com (G.F.); tommasocorsalini@gmail.com (T.C.); saverio.capodiferro@uniba.it (S.C.); massimo.corsalini@uniba.it (M.C.)

**Keywords:** teeth impaction, pediatric dentistry, pediatric surgery

## Abstract

**Background**: Dental eruption pathways could be influenced by several factors, both general and local, with different prevalence and morbidity. This study aims to report our experience of pediatric impacted teeth due to local factors, with the exclusion of the third molars, illustrating their prevalence, diagnostic and therapeutic pathways, and treatment outcomes. **Methods**: The inclusion criteria were minor age (<18 years) and the presence of impacted teeth due to a local cause, excluding wisdom teeth. The complete diagnostic and therapeutic procedures and their outcomes were described. The relationship between the treatment and the outcomes was assessed with a chi-square test. **Results**: One hundred twelve patients with a single impaction were included in the study. The local causes of single impaction were: 63 odontogenic cysts (57%), 24 supernumerary teeth (21%), 17 odontogenic tumors (15%), and eight primary bone lesions (7%). During the follow-up period, 83 teeth erupted spontaneously 12–36 months following surgery (74%), 12 were extracted during surgery (11%), and 17 needed orthodontic traction to achieve their aesthetic and functional position (15%). The relationship between mini-invasive surgery and spontaneous eruption was significant (*p* < 0.00001). **Conclusions**: Within the limitations of this study, mini-invasive surgical treatment preceded by a correct diagnosis may lead to a spontaneous eruption of permanent teeth, avoiding further orthodontic intervention and premature loss of permanent teeth.

## 1. Introduction

Dental eruption is a physiological process where a tooth moves from its initial developmental position towards its functional position, emerging from the alveolar process to occlude with its antagonist for the correct development of the occlusion and craniofacial complex [1].

However, several local or genetic factors could disrupt the physiological eruption procedure.

Some genetic conditions and syndromes involving eruption failure have been described, such as cleidocranial dysplasia, Gardner syndrome, osteoglophonic dwarfism, regional odontodysplasia, oculodental syndrome, Rutherfurd type, Nance–Horan syndrome, Cherubism, Albers-Schönberg osteopetrosis, McCune–Albright syndrome, hypodontia-dysplasia of nails syndrome, osteopetrosis, mucopolysaccharidosis, and GAPO syndrome [2].

Among these, special focus has been placed on cleidocranial dysplasia patients because the paracrine signal for bone remodeling could lead to incomplete tooth eruption [3]. Even if most of the eruption defects belong to genetic syndromes, they can also be non-familial and caused by sporadic mutation, as seen in the primary failure of eruption and cleft lip and palate [2]. In this condition, the localized failure of eruption of permanent teeth has no association with any other systemic involvement.

Besides the syndromic and sporadic causes, which are considered rare occurrences, the most common cause that prevents physiological eruption is local interference. Trauma, supernumerary teeth, odontogenic tumors, odontogenic and non-odontogenic cysts, and primary bone lesions could stop the physiological eruption pathways, resulting in a single tooth impaction. Other aspects still require further research [4].

Odontomas are the most prevalent cause of local impaction [5]. Odontomas could be classified as compound and complex, depending on their histological architecture. They primarily affect the anterior maxilla, leading to an impaction of the second sextant (teeth from 1.3 to 2.3), followed by the posterior mandible (fourth and sixth sextant). Besides odontomas, another common cause of single tooth impaction is the “mesiodens”, which is a supernumerary tooth associated with the maxillary central incisor [6]. Usually, it leads to the impaction of the 1.1 or 2.1 permanent teeth. Treatment consists of surgical extraction and then waiting for the spontaneous eruption of the impacted teeth (if retained) or orthodontic extrusion (if impacted). The last common cause is ankylosis of the deciduous tooth, which is diagnosed by the absence of its periodontal space on X-rays. The tooth must be extracted as soon as possible to promote the spontaneous eruption of the permanent tooth [7].

Diagnosis of impaction typically involves a straightforward clinical and radiographic assessment. When collecting the medical history, the patient or their parents may report the absence of a tooth beyond the expected physiological timeframe, particularly if the contralateral tooth has erupted. This observation is subsequently confirmed through clinical evaluation. Radiographic exams, such as a dental X-ray and orthopantomography, reveal the tooth in question in its initial developmental position in the alveolar process, at different distances from the oral cavity. Teeth are classified as “retained” when the roots are immature, whereas they are considered “impacted” if the roots are fully developed and the dental apex is physiologically closed. Impaction of permanent teeth (excluding third molars) is a frequent phenomenon, with a reported prevalence ranging from 2.9% to 13.7%. The most frequently impacted teeth, aside from the third molars, with varying incidence rates, are the maxillary canines, followed by mandibular premolars and maxillary incisors, regardless of gender [8].

Treatment requires multidisciplinary management and should aim for a full recovery of the impacted tooth. A pediatric dentist or an oral surgeon must remove the local etiopathogenetic cause when present, and finally, where necessary, an orthodontist could move the tooth to its esthetic and functional position. However, when this is impossible, and the impacted tooth needs to be extracted, an adhesive fixed prosthesis and implant-supported solution could be considered as viable alternatives [9].

Moreover, the existence of a follicle adjacent to the crown of an impacted tooth is commonly associated with the formation of cysts and tumors, such as dentigerous cysts, keratocystic odontogenic tumors, and ameloblastoma, which may arise from odontogenic epithelial rests [1].

Although many studies have examined this topic, consensus on treatment protocols remains limited; with our work, we aimed to overcome the limitations of the existing literature by applying well-defined inclusion criteria and standardizing both diagnostic methods and postoperative follow-up timing.

This retrospective study is intended to report our experience with impacted teeth (excluding the third molars). We aimed to describe the prevalence and the cause of eruption disturbances and to evaluate the outcome of different types of treatment, through a long-term follow-up.

## 2. Materials and Methods

This retrospective observational study was conducted in the Pediatric Dentistry department of the Pediatric Hospital “Giovanni XXIII”, Bari, Italy. Our analysis involved patients with impaction of permanent or deciduous teeth due to a local factor; therefore, we focused on impaction related to the presence of supernumerary teeth, odontogenic cysts, odontogenic tumors, and primary bone lesions. This study was retrospectively carried out on a database that collects data from medical records. The database included: name of patient, age, sex, tooth impacted, local cause which prevented the normal eruption, surgical approach, and outcome and follow-up. Manual record screening was performed on the database, based on the following inclusion and exclusion criteria, resulting in a consecutive series over 10 years of public service, in the period from 2012 to 2022.

The inclusion criteria were patients aged from 0 to 18 years, patients who showed single teeth impaction (defined as the delay of eruption exceeding >6 months from the normal eruption timetable), healthy patients who could be subjected to oral surgery procedures, and patient (or their parents) who signed the informed consent. We excluded third molars, impacted canines for orthodontic reasons, impacted or retained teeth related to systemic factors or trauma, and patients who did not complete the follow-up. Third molar impaction was excluded because of its high rate, and its common cause (malposition or absence of space). Moreover, the literature on third molar impaction is too wide, so we decided that its inclusion was not necessary.

Written informed consent using a standard institutional consent form was obtained from the patient’s parents for each diagnostic and therapeutic step, including collecting data for scientific purposes. In particular, the standard institutional consent form allowed the clinician to treat the patient for their actual disease and to keep the data (excepting the personal identification information) for eventual further scientific purposes. Without written consent, the clinicians were not allowed to treat the patient, regardless of the scientific purposes.

The diagnostic and therapeutic management reflects common patterns of care that were performed regardless of the study, but exclusively for therapeutic purposes. No protocol or study interventions were conducted. For this reason, and because it is a retrospective study carried out exclusively on a database of medical records, no ethical approval was required under local regulations.

The process was the same for all the patients. An initial introduction and interview was carried out to collect sex, age, and medical and dental information. Often, during the same meeting or a new meeting, one or more pediatric dentists or general dentists performed a clinical visit. Then, each patient underwent a first-level dental X-ray investigation: orthopantomography (OPT) of the dental arches or X-rays to find the missing tooth and any possible local factor preventing its physiological eruption. A Computed Tomography Cone Beam (CBCT) was performed to better visualize the local cause’s three-dimensional position, its extent, and its relationship with the impacted/retained tooth. Radiological images were obtained using Kavo OP 3D (Kavo, Biberach, Germany), with a mean radiation dose of 143.2 mGycm. This diagnostic pathway allowed for the planning of the correct and mini-invasive or extensive surgical approach required by the specific patient, which was performed by an expert pediatric oral surgeon. The sample was then sent for histopathological examination after excision. No orthodontic traction was applied immediately after the surgical step, and spontaneous eruption was allowed to occur. Mini-invasive surgery was performed on odontogenic cysts and supernumerary teeth, while extensive surgery was performed on odontogenic tumors and primary bone lesions.

**Figure 1:**
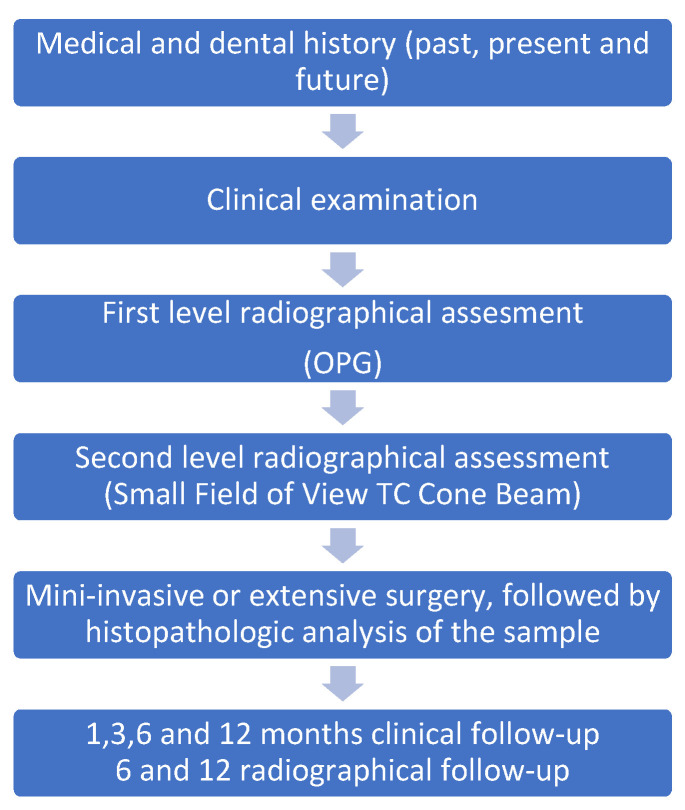
Diagnostic-therapeutic procedures.

Clinical follow-up was established at 1, 3, 6, and 12 months for the first year, and yearly from the second year. Radiographic follow-up was scheduled for 6 and 12 months with OPT and CBCT. The diagnostic-therapeutic procedures are listed below (Figure 1).

A chi-square test was performed to assess the relationship between the treatment option (mini-invasive surgery vs. extensive surgery) and the outcome (spontaneous eruption vs. non-spontaneous eruption). Statistical analysis was conducted with Stata^®^, version 13.0 (Stata Corp., College Station, TX, USA).

## 3. Results

Overall, 167 patients were considered in this study; 55 were excluded because they did not complete the follow-up. Consequently, a total of 112 patients participated in the study, comprising 60 males (54%) and 52 females (46%). All of them presented with a single impaction due to local issues. Their age was in a range from 6 to 17 years old, with a mean of 10.9 ± 2.7 years.

Impaction was observed primarily in the permanent dentition: 103 permanent teeth (92%) vs. nine deciduous teeth (8%).

The localization was slightly more frequent in the maxilla (65 cases, 58%) compared to the mandibular localization (47 cases, 42%). For the primary dentition, the impaction affected five maxillary canines (5%), three maxillary molars (3%), and one mandibular canine (1%). For the permanent dentition, the maxillary teeth affected included 13 central incisors (12%), 14 lateral incisors (13%), 29 canines (25%), and nine premolars (8%). The mandibular teeth affected included nine central incisors (8%), 17 canines (14%), and 12 molars (11%). Therefore, the distribution of affected teeth was predominantly in the anterior region of both arches (79%), followed by the latero-posterior region of the mandible (21%).

The local factors that prevented the physiological eruptive pathway were: 63 odontogenic cysts (57%), 24 supernumerary teeth (21%), 17 odontogenic tumors (15%), and eight primary bone lesions (7%).

Mininvasive surgery was performed on 87 patients (78%) with diagnosis of odontogenic cysts and supernumerary teeth, while extensive surgery was performed on 25 patients (22%) with a diagnosis of odontogenic tumors and primary bone lesions. The mean follow-up period was 20.6 ± 5 months, with a minimum of 12 months and a maximum of 34 months.

During the follow-up period, we observed that 83 teeth erupted spontaneously following surgery (74%) after 12–36 months in 63 patients with odontogenic cysts, 13 patients with mesiodens, and seven patients with odontogenic tumors. No patients with primary bone lesion showed spontaneous eruption.

Of the remaining 29 teeth, 12 were extracted during the surgery (11%) to minimize the chance of recurrence, while 17 required orthodontic traction to reach their aesthetic and functional position (15%).

A synoptic table of the results with descriptive statistics is shown below (Table 1):

The chi-square test showed a significant correlation between mini-invasive surgery and the spontaneous eruption of the treated teeth (*p* < 0.00001. Significant at *p* < 0.05), reported as follows (Table 2).

## 4. Discussion

In this study, we reported our institutional experience concerning the prevalence, etiological causes, and therapy outcomes of pediatric teeth impaction due to local factors. We discuss our results and future perspectives as follows. Tooth impaction related to the presence of an obstacle in the typical path of eruption during the first decades of life seems to be an interesting topic for the pediatric dentist. In the literature, we found a large variety in its prevalence, ranging from 2.9% to 18.8% [10,11,12]. Overall, most of the studies agree on the low prevalence of this occurrence: Dorotheu et al. [3] reported a prevalence of 6.3%, and Uslu et al. [11] and Gupta et al. [13] also reported a lower incidence of impaction (2.9% and 3.74%, respectively). Impaction of a deciduous tooth is uncommon and is more likely to be associated with the presence of a supernumerary tooth or an odontoma [11], especially in the frontal sector, but the exact cause and the significance of multiple impacted supernumerary teeth remain an enigma.

### 4.1. Relationships Between Odontogenic Cysts and Eruption Disorders

Unerupted teeth are strongly associated with the presence of odontogenic cysts [14,15]. Consistent with several authors [16] who reported odontogenic cysts as the most common orofacial lesion among children, we found that odontogenic cysts were prevalent in more than half of the cases of our sample (57%). The most reported cysts in pediatric patients are dentigerous cysts, with a frequency of 1.44 cysts in every 100 unerupted teeth, according to Rohilla et al. [17]. In a recent meta-analysis, Nahajowski et al. reported a 62% spontaneous eruption of impacted teeth due to an odontogenic cyst [18]. In our sample, we report 100% of patients with an odontogenic cyst who showed a spontaneous eruption of the permanent tooth, higher than in previous literature. This result can be explained by the fact that each patient with a preoperative diagnosis of odontogenic cyst was treated with mini-invasive surgery, emphasizing the importance of this type of surgery.

### 4.2. Relation Between Supernumerary Teeth and Tooth Impaction

Early diagnosis of a supernumerary tooth is essential to reduce complications, comorbidities, and complex orthodontic treatments. Supernumerary teeth are usually single teeth (mesiodens), with a prevalence range from 0.2% to 3% [19]. On the other hand, multiple supernumerary teeth are related to systemic conditions or syndromes [20]. Authors reported only a few cases of multiple supernumerary teeth that were unrelated to syndromes or systemic conditions [21,22]. In our sample, supernumerary teeth represented 21% of local factors preventing a physiological eruption. All patients underwent mini-invasive surgical extraction and waited for the natural eruption of the underlying tooth. In fact, the most suggested therapy consists of surgical removal of the supernumerary tooth, which often results in the spontaneous eruption of the impacted tooth, ranging from 39.7% to 89.4% of cases [23]. Likewise, 13 of 24 mesiodens (54%) resulted in a spontaneous eruption.

### 4.3. Relations Between Odontogenic Tumors and Tooth Impaction

Almost half of odontogenic tumors are odontomas [24], which makes it the most common odontogenic tumor. In our sample, odontomas represented 15% of the local causes of impacted teeth. Sella Tunis et al., in their meta-analysis, concluded that 57% of odontomas generated symptoms such as delayed eruption of teeth, swelling, pain, inflammation, and infection [25]. Odontomas are typically discovered during routine radiographic exams. A later diagnosis could result in damage to the adjacent teeth, so early detection is associated with a better prognosis for the impacted teeth [26]. Spontaneous eruption of teeth impacted due to odontomas occurs in 45% of cases after the obstacle is removed [4]. In our sample, spontaneous eruption occurred in seven patients with odontomas out of 17 (41%), slightly lower than the percentage reported in the literature.

### 4.4. Relationship Between Primary Bone Lesions and Tooth Impaction

Oral primary bone lesions are a rare entity that include osteoid osteomas, giant cell tumors, ossifying fibromas, ameloblastic fibromas, ameloblastic fibro-odontomas, and aneurysmal bone cysts [27]. Le Donne et al. (2019) reported a case of an impacted mandibular canine in an 11-year-old boy, who was affected by an ossifying fibroma [28]. Gadre et al. reported a single case of a wisdom tooth impacted under a large osteoid osteoma [29]. In their interesting multicenter study, Roza et al. reported a rate of impaction of 40% for ameloblastic fibromas and up to 63% for ameloblastic fibro-odontomas in an adult cohort [30]. No studies evaluate spontaneous eruption after the removal of these lesions. Despite the low prevalence previously described, and the few studies that assess teeth impaction in primary bone lesions, we found eight cases (7% of the entire sample) of single impaction related to primary bone lesions. No patients showed a spontaneous eruption (as primary bone lesions required extensive surgery).

### 4.5. Treatment Outcomes

According to the literature, the range of spontaneous eruption following the removal of an obstruction for a permanent tooth is between 39.7% and 89.4% [31]. A similar percentage was found in our 10-year retrospective study: 83 teeth (74%) were fully recovered after a spontaneous eruption. Factors that influence the eruption of an impacted permanent tooth due to an obstacle are the position of the tooth, the state of root development, the presence of root dilaceration, the amount of space needed for its placement in the arch, the proximity of the obstacle to the bud of the dental follicle, and the age of the patient. The heterogeneity of factors affecting tooth eruption is so numerous that it is impossible to generalize, as noted in the literature.

### 4.6. Limitations and Future Directions

This study acknowledges certain limitations. This study aims to contribute data on this topic, addressing the gap in similar studies in the existing literature. Although a few studies involved more patients, they considered a small sample of patients. However, the lack of literature leads to an inconsistent comparison. Potential selection bias, the quality of the retrospective data, and the generalizability of findings are other weaknesses of this study. Drawing definitive conclusions about the correct therapy for teeth impaction due to a local factor is not possible with this study. Further studies with controls and larger samples are required, along with a correct meta-analysis of the current literature, to establish the gold standard.

## 5. Conclusions

This paper reports our institutional experience, describing the diagnostic and therapeutic pathways of such a common occurrence as teeth impaction due to a local obstruction. Mini-invasive surgical treatment, preceded by correct timing and a proper diagnosis, is related to a spontaneous eruption of permanent teeth. Moreover, the pediatric dentist must be aware of this occurrence, which plays a pivotal role in early diagnosis and treatment and is often performed by a pediatric oral surgeon. This stresses the importance of multidisciplinary collaboration, which is mandatory in these complex cases.

## Figures and Tables

**Table 1:** Synoptic table of results.

Results	Number (Percentage)	Mean ± SD	Standard Error	95% Conf. Interval
**Sex**			5.30	49.5–70.5
Male	60 (54%)			
Female	52 (46%)			
**Age**		10.9 ± 2.75 Years		6 (Min.)–17 (Max.)
**Dentition**			2.89	97.3–108.7
Permanent	103 (92%)			
Deciduous	9 (8%)			
**Localization**			5.25	54.6–75.4
Mandibulary	47 (42%)			
Maxillary	65 (58%)			
**Local Causes**				
Cysts	63 (57%)		5.27	52.6–73.5
Supernumerary teeth	24 (21%)		4.36	15.4–32.6
Odontogenic tumors	17 (15%)		3.81	9.4–24.6
Primary bone lesion	8 (7%)		2.73	2.6–13.4
**Type of Surgery**			4.43	54.6–75.4
Mini-invasive	87 (78%)			
Extensive	25 (22%)			
**Follow-up**		20.6 ± 5.04 Months		12 (Min.)–34 (Max)
**Outcome**			4.66	73.8–92.2
Spontaneous eruption	83 (74%)			
Orthodontic treatment/extraction required	29 (26%)			

**Table 2:** Chi-square test analysis.

Type of Eruption	Mini-Invasive Surgery	Extensive Surgery	*p*-Value (Significant at *p* < 0.05)
Spontaneous eruption	76	7	
Non-spontaneous eruption	11	18	
Total	87	25	*p* < 0.00001

## Data Availability

The data presented in this study are available on request from the corresponding author due to privacy reasons..
